# Near-Infrared Spectroscopy for Mapping of Human Meniscus Biochemical Constituents

**DOI:** 10.1007/s10439-020-02578-x

**Published:** 2020-07-27

**Authors:** Juho Ala-Myllymäki, Tommi Paakkonen, Antti Joukainen, Heikki Kröger, Petri Lehenkari, Juha Töyräs, Isaac O. Afara

**Affiliations:** 1 Department of Applied Physics, University of Eastern Finland, Kuopio, Finland; 2 Institute of Biomedicine, University of Eastern Finland, Kuopio, Finland; 3 Department of Orthopaedics, Traumatology and Hand Surgery, Kuopio University Hospital, Kuopio, Finland; 4 Kuopio Musculoskeletal Research Unit, Institute of Clinical Medicine, University of Eastern Finland, Kuopio, Finland; 5 Cancer and Translational Medicine Research Unit, Medical Research Center Oulu, University of Oulu and Oulu University Hospital, Oulu, Finland; 6 School of Information Technology and Electrical Engineering, The University of Queensland, Brisbane, Australia; 7 Diagnostic Imaging Center, Kuopio University Hospital, Kuopio, Finland

**Keywords:** Meniscus, Spectroscopy, Near-infrared, Regression analysis, Biomedical technology

## Abstract

Degenerative changes in meniscus are diagnosed during surgery by means of mechanical testing and visual evaluation. This method is qualitative and highly subjective, providing very little information on the internal state of the meniscus. Thus, there is need for novel quantitative methods that can support decision-making during arthroscopic surgery. In this study, we investigate the potential of near-infrared spectroscopy (NIRS) for mapping the biochemical constituents of human meniscus, including water, uronic acid, and hydroxyproline contents. Partial least squares regression models were developed using data from 115 measurement locations of menisci samples extracted from 7 cadavers and 11 surgery patient donors. Model performance was evaluated using an independent test set consisting of 55 measurement locations within a meniscus sample obtained from a separate cadaver. The correlation coefficient of calibration (*ρ*_training_), test set (*ρ*_test_), and root-mean-squared error of test set (RMSEP) were as follows: **water** (*ρ*_training_ = 0.61, *ρ*_test_ = 0.39, and RMSEP = 2.27 percentage points), **uronic acid** (*ρ*_training_ = 0.68, *ρ*_test_ = 0.69, and RMSEP = 6.09 basis points), and **hydroxyproline** (*ρ*_training_ = 0.84, *ρ*_test_ = 0.58, and error = 0.54 percentage points). In conclusion, the results suggest that NIRS could enable rapid arthroscopic mapping of changes in meniscus biochemical constituents, thus providing means for quantitative assessment of meniscus degeneration.

## Introduction

The menisci are fibrocartilaginous semilunar structures located in the knee joint between the tibial plateau and femoral condyles. Meniscus is composed of water (70–75%), collagen (20–22%), and glycosaminoglycan (0.6–0.8%).^12^ The main functions of the menisci are load transmission, shock absorption, and stabilization of the knee joint. Vascularization of the human meniscus changes drastically with age.^20^ Infant menisci are completely vascularized, but vascularity withdraws to the outer 10–30% portion of the menisci with aging.^5^,^20^ Vascularity of the meniscus is strongly related to its regenerative capabilities.^5^

Meniscus functional properties have been shown to deteriorate due to aging and injury.^11^,^12^ Traumatic injuries may occur during sporting events, where menisci are affected by high peak stresses. On the other hand, the meniscus of elderly people may be injured even from normal day to day activities due to age-related degeneration. When meniscus degenerates, its water content increases, while the collagen and glycosaminoglycan contents decrease,^12^,^26^ thus initiating either partial tears, which may develop into meniscal cysts, or ruptures. If the injury extends to the meniscal root or attachment points, it may result in meniscal extrusion. Although there is a large variation in the frequency and types of meniscal tears, the general consensus is that degenerative tears are more common.^16^ The location of the injury is vital when selecting the optimal treatment method.^18^,^29^ Conservative treatment is typically selected if the region of injury is in the avascularized area of meniscus. However, if the injury is in the vascularized zone, arthroscopic repair surgery may be suitable, as the meniscus regenerative capability is better in this zone.^29^

If meniscal defects are suspected, initial diagnosis is performed via clinical evaluation based on patient history, physical examination, and radiography. McMurray’s test is a typical physical examination performed on patients prior to imaging. If there is pain or popping sound during this test, it is an indication of meniscal injury. Plain radiography is unable to visualize the menisci, but it can show joint attrition and degenerative extrusion, which are key factors for degenerative meniscal injury. Following initial diagnosis, further investigation may be undertaken using magnetic resonance imaging (MRI), which is currently the gold standard imaging method for soft connective tissues of the knee joint. MRI is an effective imaging modality due to its noninvasiveness; however, its availability is limited. Furthermore, there are still challenges in identifying certain meniscal injuries in clinical MRI images, for example tears located in the posterior horn of the lateral meniscus.^17^,^28^ Upon validation of initial diagnosis, arthroscopic repair is often conducted, especially if mechanical symptoms are prominent. Meniscus is visually and mechanically evaluated during arthroscopy, which is the current clinical gold standard for repair of joint connective tissue injuries. The advantage of arthroscopic surgery is that repair (suturing or partial meniscectomy) can be performed simultaneously with the evaluation of the menisci. However, arthroscopic evaluation is qualitative and highly subjective, with poor inter- and intra-observer reliability.^6^,^8^,^23^ Furthermore, it does not provide information on internal structure and composition or the microvascular status of meniscus. Hence, quantitative, and objective arthroscopic techniques for evaluation of meniscus integrity are urgently needed.

Near-infrared spectroscopy (NIRS) is a method based on molecular overtone and combination vibrations. NIRS is sensitive to O–H, N–H, C–H, and S–H bonds, which are common in biological materials. NIRS has already been successfully applied for assessment of several connective tissues.^2^,^13^,^21^,^22^,^24^,^25^,^27^,^30^ So far, we have successfully demonstrated relationships between the near-infrared spectral response of human meniscus and its constituents and biomechanical properties.^3^,^4^ In this study, we hypothesize that NIRS can be adapted for mapping, and potentially visualizing changes in the composition of human meniscus. To test this hypothesis, we developed and applied partial least squares regression (PLSR) models for mapping the water, collagen, and glycosaminoglycan contents of human meniscus.

## Materials and Methods

### Sample Collection

Menisci samples were obtained from two sources. For the first set of samples, lateral and medial menisci were obtained from both knees of human cadavers (*N*_cadaver_ = 8) from Kuopio University Hospital (Approval number: 58/2013 by The Research Ethics Committee of the Northern Savo Hospital District). The second set of samples were collected from patients undergoing total knee endoprosthesis surgeries (*N*_donor_ = 11) at Oulu University Hospital (Approval number: 78/2013 by Ethical committee of North Ostrobothnia’s hospital district).

### Near-Infrared Spectroscopy

Diffuse reflectance NIRS measurements were acquired in a dark room using an optical spectrometer consisting of a light source (AvaLight-HAL-S-Mini, Avantes BV, Netherlands) and detector (AvaSpec-NIR256-2.5-HSC, Avantes BV, Netherlands) with spectral range 1000–2500 nm and resolution of 6.55 nm. The optical spectrometer was equipped with a custom-made arthroscopic probe (diameter = 3.25 mm) consisting of 114 optical fibers (diameter = 100 *μ*m) inside the steel casing of the probe. 100 of the fibers were used for transmitting the light from the light source to the sample, and 14 fibers were used for receiving the diffuse reflected light from the sample to the detector. From the 14 receiving fibers, 7 were connected to the detector used in this study, and the remaining 7 fibers were connected to a UV/Visible detector (300–1100 nm), not used in this study. The optical spectrometer integration time for a single acquisition was 16 ms, and a single measurement consists of 100 coadded scans.

Prior to measurements, samples were divided into training and test sets. Training set (*n*_training_ = 115) consisted of measurements acquired from the anterior, central, and posterior locations of the menisci samples, with one cadaver meniscus left out. The test set (*n*_test_ = 55) consisted of measurements acquired from 55 locations on the right medial meniscus of the cadaver left out from the training set.

Prior to NIRS measurements on the training set, the samples stored in − 20 °C were thawed at room temperature for at least 1 h and the phosphate buffered saline (PBS) with inhibitors of proteolytic enzymes: Ethylenediaminetetraacetic acid disodium salt dihydrate (EDTA; VWR International, Fontenay-sous-Bois, France) and benzamidine hydrochloride hydrate (Sigma-Aldrich Corp., St. Louis, Missouri, USA) was warmed up to controlled room temperature (20–25 °C). Subsequently, the samples were placed on top of a black rubber base in a container filled with PBS. Each anatomical location (anterior, central, and posterior) was measured three times with probe realignment between measurements. The representative spectrum, later used in partial least squares regression (PLSR) analysis, was obtained as the average of the three spectral measurements acquired at each location.

The mapped test set sample stored in − 20 °C was also thawed at least 1 hour at room temperature. Sample was positioned on top of a gridded stage (grid size = 3.4 × 3.4 mm). Near-infrared spectra were measured three times at each grid location on the sample. During measurement, adjacent locations of the meniscus were kept moist by wrapping them with PBS-soaked tissue towels.

### Biochemical Measurements

Approximately 1 mm wide full-thickness specimens were extracted radially adjacent to each of the NIRS measurement locations for biochemical analysis. First, the wet weight (WW) of each specimen was measured. Subsequently, the specimens were freeze-dried to determine the matrix dry weight and calculate the water content. The specimens were digested for 16 h in papain (1 mg ml^−1^) and 150 mM sodium acetate including 5 mM Cys–HCl and 15 mM EDTA at 60 °C and pH of 5.8.^14^ The papain-digested specimens were cooled and hydrolyzed, and the hydroxyproline (HP) content was determined spectrophotometrically.^9^ Uronic acid (UA) content was quantified from ethanol-precipitated residue of the digested specimens.^7^ The contents were normalized to the WW of the specimen to compensate for variation in specimen sizes.

### Spectral Preprocessing and Multivariate Analysis

Spectral preprocessing was performed using nippy (https://github.com/uef-bbc/nippy), a custom program written in Python and developed in our research group. The program creates multiple combinations of preprocessing options based on selected input options, such as spectral range, derivative pre-treatment, scatter correction, etc. The spectral ranges used in this study were selected to be 1060–1172 nm, 1211–1366 nm, 1560–1840 nm, and combinations of these ranges. The spectral ranges of 1000–1060 and 1172–1211 nm were left out of analysis due to high noise in specified regions and the spectral ranges of 1366–1560 and 1840–2500 nm were left out of analysis due to saturation in the spectra. The different scatter correction method applied in spectral preprocessing include standard normal variate (SNV), robust normal variate (RNV), and multiplicative scatter correction (MSC). Following scatter correction, spectral smoothing was performed using Savitzky–Golay (SG) filter with different window sizes (5, 7, 9, 11, 13, 15, 17, and 19), polynomial orders of (2 and 3), and derivative orders of (0, 1, and 2).

PLSR analysis was performed using the LibPLS package^15^ (www.libpls.net) in MATLAB (MathWorks, Natick, Massachusetts). The training set spectral data (predictors) were correlated with the biochemical constituents (response) using nonlinear iterative partial least squares (NIPALS) algorithm, which maximizes covariance between the predictors and the response variables. Monte Carlo cross-validation (MCCV) technique with 1000 iterations and data selection ratio of 90% was used to estimate the root-mean-square error of cross-validation (RMSECV) and to determine the optimal number of PLSR components. Cross-validation was selected as the method of choice due to limited number of samples. In each iteration, MCCV randomly creates a train-validation split, where the model is trained using 90% of the available data. The estimated RMSECV values from each iteration are then averaged, thus providing a realistic evaluation of the model performance to minimize potential sample dependent bias in test set. In order to validate the models’ performance, root-mean-square error of calibration (RMSEC) and Spearman’s rank correlation coefficient *ρ*_training_ were calculated for the training set, while root-mean-square error of prediction (RMSEP) and Spearman’s rank correlation coefficient *ρ*_test_ were calculated for the test set. Percent point (pp) and basis point (bp) units are used to present RMSECV, RMSEC, and RMSEP as they are based on arithmetic difference of percentage values (% of WW).

Outlier detection was performed using Monte Carlo sampling method with 2500 iterations and data selection ratio of 75%. After detection of potential outliers, comparison between base PLSR model and PLSR models developed with different variable selection methods was performed. The variable selection methods applied include uninformative variable elimination (UVE), random frog (FROG), competitive adaptive reweighted sampling (CARS), moving window partial least squares (MWPLS), and iteratively retaining informative variables (IRIV).

### Spatial Mapping of Biochemical Constituents in Meniscus

After prediction of the test meniscus constituents using the optimal models, the target variables from the 55 measurement points were formatted into a 9 × 14 matrix, with each element (pixel) corresponding to a measurement location on the grid. Since spectral measurement could not be acquired from edges of the meniscus due to probe only partially covering the sample, missing grid locations were calculated as mean of neighboring locations to enable better visualization of meniscus constituents. The resulting map was centered within a 20 × 20 matrix of zeros and linear interpolation (spacing = 0.01) was used to increase the matrix dimension to 1901 × 1901 pixels, thus improving the spatial resolution of the map. The map was then imaged in to visualize distribution of the target property. Linear interpolation was performed in MATLAB using interp2 function.

## Results

The optimal preprocessing parameters for the spectra varied depending on the reference (Table 1). No sample was detected as an outlier; thus, all measured samples were retained for analysis. The optimal PLSR models with and without the use of variable selection methods are listed below (Table 2). The base model (i.e., without variable selection) was best for predicting meniscus water content, while the best models for predicting UA and HP were optimized using IRIV and FROG variable selection methods, respectively. The test set RMSEP relative to range of reference parameter were 14.7% for water, 24.7% for UA, and 20.3% for HP.

**Table 1:** The wavelength ranges and preprocessing methods for optimizing the correlation between NIRS spectra and meniscus composition based on PLSR.

Reference	Wavelength (nm)	Scatter correction	SG smoothing		
			Window size	Polynomial order	Derivative order
Water	1060–1172 1211–1366 1560–1840	SNV	17	2	0
Uronic acid	1060–1172 1211–1366	RNV	13	2	0
Hydroxyproline	1060–1172 1211–1366 1560–1840	SNV	13	2	0

**Table 2:** PLSR models trained on the optimally preprocessed spectral data for each reference property with and without variable selection.

Water	*ρ*_training_ (–)	RMSEC (pp)	RMSECV (pp)	*ρ*_test_ (–)	RMSEP (pp)	*n*_comp_ (–)
**Base**	**0.61**	**2.15**	**2.49**	**0.39**	**2.27**	**6**
UVE	0.57	2.24	2.39	0.27	2.69	4
FROG	0.66	2.05	2.39	0.32	3.23	7
CARS	0.49	59.9	2.82	− 0.11	57.6	1
MWPLS	0.57	2.23	2.50	0.29	4.62	6
IRIV	0.67	2.06	2.26	0.34	2.48	6

UA	*ρ*_training_ (–)	RMSEC (bp)	RMSECV (bp)	*ρ*_test_ (–)	RMSEP (bp)	*n*_comp_ (–)
Base	0.74	3.64	5.12	0.71	6.88	13
UVE	0.69	3.90	4.45	0.67	7.52	11
FROG	0.75	3.56	4.82	0.57	7.29	13
CARS	0.74	23.2	5.44	0.47	29.0	1
MWPLS	0.64	4.12	5.27	0.38	5.96	11
**IRIV**	**0.68**	**3.89**	**4.37**	**0.69**	**6.09**	**10**

HP	*ρ*_training_ (–)	RMSEC (pp)	RMSECV (pp)	*ρ*_test_ (–)	RMSEP (pp)	*n*_comp_ (–)
Base	0.85	0.26	0.49	0.62	0.63	17
UVE	0.78	0.32	0.42	0.32	1.07	13
**FROG**	**0.84**	**0.26**	**0.42**	**0.58**	**0.54**	**16**
CARS	0.80	4.44	0.53	0.60	3.85	6
MWPLS	0.68	0.39	0.53	0.38	1.07	8
IRIV	0.80	0.32	0.42	0.27	0.85	13

Bold highlighted texts indicate the best model for each reference property

**Figure 1:**
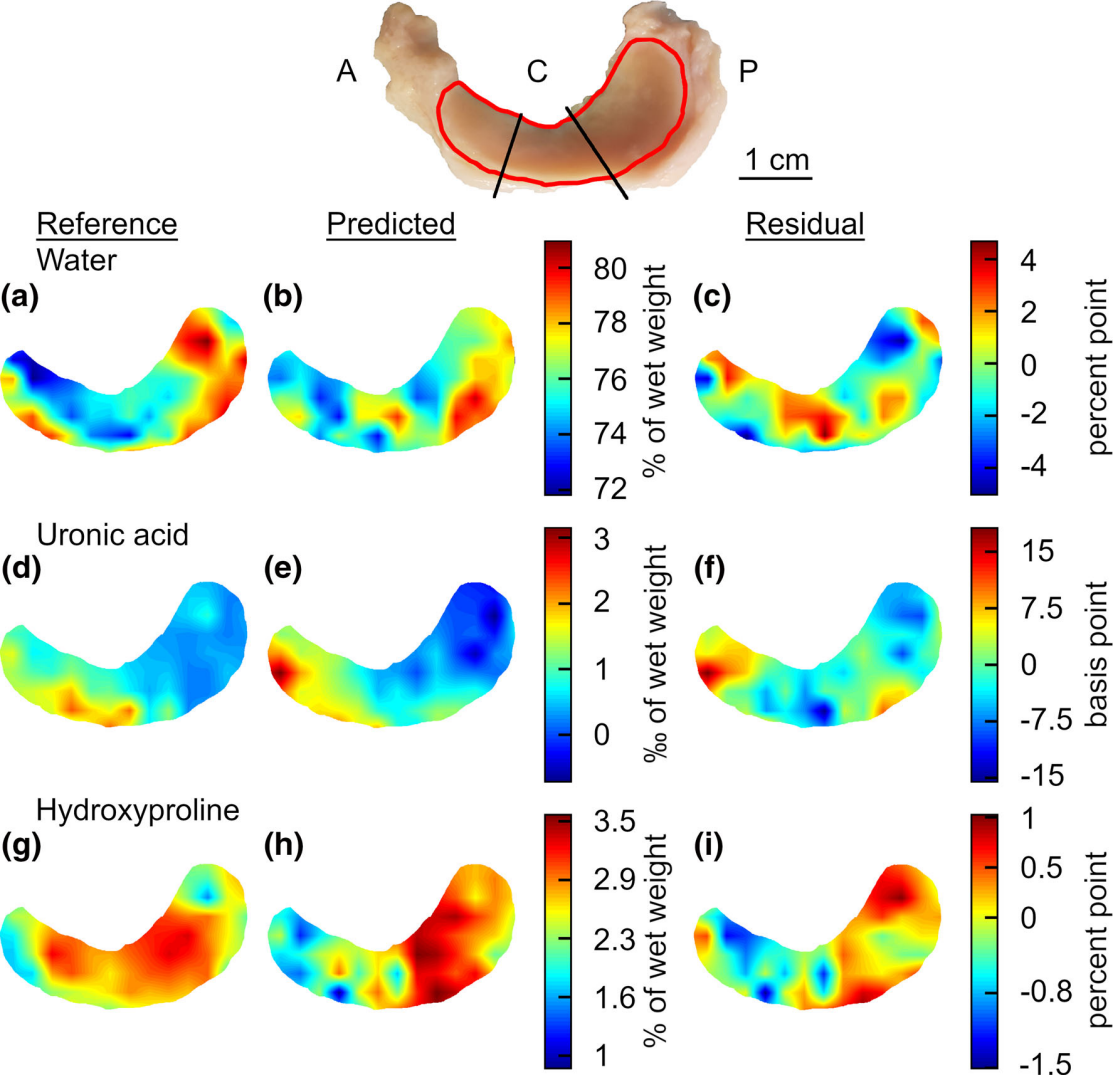
Independent meniscus sample left out as test set meniscus is presented at the top corner of the figure with its anatomical regions (A = anterior, C = central, and P = posterior). Edges of the colormaps are highlighted with red line on top of the meniscus sample. Side by side comparison of the distribution of the test set meniscus water ((a) measured, (b) predicted), uronic acid ((d) measured, (e) predicted), and hydroxyproline ((g) measured, (h) predicted) content. Prediction error maps are presented for each biochemical property in (c), (f), and (i), respectively.

Visualization of the biochemical composition map of the test set meniscus based on measured and PLSR-predicted water, uronic acid, and hydroxyproline values, together with the localized errors, are presented in Fig. 1. The scale bar in Fig. 1 was determined by placing a ruler next to the meniscus sample when acquiring the image of the sample prior to NIRS measurements. The model for predicting meniscus water content was the most accurate in terms of relative errors. The error distribution of this model from pairwise comparison of the measured and predicted values per locations were as follows: min = 0.08%, max = 6.52%, mean = 2.41%, and median = 2.48%. The models for predicting UA had large relative errors: min = 0.80%, max = 488.59%, mean = 91.78%, and median = 54.08%, while the model for estimating HP from the spectral data was relatively good, with only few measurement locations having large error. The distribution of this model’s errors is as follows: min = 0.41%, max = 65.55%, mean = 17.07%, and median = 13.98%.

## Discussion

In this study, we demonstrate, for the first time, the relationship between meniscus NIRS spectral data and its biochemical composition using parts of the second (1100–1350 nm) and third (1600–1870 nm) optical windows. In addition, we also demonstrated the capacity of NIRS for mapping the biochemical composition of human meniscus. In an earlier study, we demonstrated that prediction of meniscus biochemical composition was possible using spectral data in the visible (400–750 nm) and first optical window (750–1100 nm) spectral ranges; however, that study was restricted to predicting meniscus composition at three specific anatomical locations^4^ (anterior, central, and posterior). Although the results between these two studies are not directly comparable, they indicate that models based on the second and third optical window could be favorable for predicting meniscus water and HP contents, while use of a model based on the first optical window could be favorable for predicting meniscus UA content. NIRS based mapping of connective tissue constituents has been previously demonstrated for bovine^1^ and equine articular cartilage.^22^

In the present study, samples were extracted from the knee joints of human cadaver subjects and patients undergoing knee surgery. Although metabolism in the samples was different at the point of extraction, all samples were metabolically inactive at the point of measurement. Also, due to the nature of meniscus, which consists of a large quantity of extracellular matrix and relatively few live cells, it is conceivable that the cadaver meniscus is, by physical properties, very similar to those of live patients. Samples were preserved as described in the Materials and Methods sections to prevent any potential alteration in the matrix constituents. As with other biological tissues, the composition and structure of meniscus are the main drivers of its functional integrity.^11^ Therefore, any alteration in meniscus water, collagen and glycosaminoglycan content will result in compromised biomechanical competence and eventually lead to tissue degeneration^12^,^26^ Thus, it is crucial to investigate the potential of NIRS to non-destructively estimate meniscus constituents via separate models, which can then be later combined into a single model for holistic evaluation of meniscus matrix integrity. This could enable creation of quantitative methods that could assist decision-making during arthroscopic surgery, potentially minimizing the effect of subjectivity on treatment outcome. It is worth noting that this is beyond the scope of the present study.

Based on the present results the choice of optimal preprocessing options and parameter of the spectral data depend on the target tissue property. This may be due to different combinations of preprocessing methods highlighting certain regions of the spectra associated with the specific tissue property. However, due to the broad and overlapping peaks in the NIRS spectral range, it is difficult to define the exact molecular features that are highlighted in each spectral region of interest. Nevertheless, water has strong absorption bands around 1400–1450 nm.^31^ Although the model for predicting meniscus water content in this study used a different spectral range from what was previously reported,^31^ it achieved low prediction errors using water peaks spectral shoulder regions.

Proteoglycans play an important function in binding water into the meniscus matrix. Proteoglycans are composed of glycosaminoglycans (GAG) attached to core proteins while GAG is composed of repeating disaccharide unit on which one of the sugars is UA. The best model for predicting UA was achieved using spectral data within the wavelength ranges of 1060–1172 and 1211–1366 nm. The performance of the best UA model was still suboptimal, possibly due to the very low concentration of UA in meniscus. Thus, even small errors in reference biochemical measurements would significantly affect the performance of the final model.

HP was selected as the proxy for meniscus collagen content as it is a fundamental part of collagen triple helical structure. HP is composed of a hydroxyl group attached to proline amino acid. The main features of different kinds of amino acids have been previously reported to be in the wavelength regions of 1650–1794 and 1840–1940 nm.^10^ The best model for predicting HP was achieved using spectral data within the wavelength ranges of 1060–1172, 1211–1366, and 1560–1840 nm. Thus, the best HP model was partially in agreement with the previously reported relevant wavelength regions. Model performance was overall satisfactory with only few measurement locations having large errors.

MRI is often used for qualitative clinical assessment of meniscus. However, MRI is also capable of providing quantitative information on meniscus composition and structure. Therefore, MRI would have been the clinical reference modality of choice to validate the present results. Unfortunately, due to resource and time constraints it was not possible to conduct MRI on the samples used in the present study. Nevertheless, the models developed for estimation of meniscus constituents from its NIRS spectral data were validated using cross-validation approach, and their performance were assessed on an independent test set.

A potential limitation of the present study is related to ensuring the proper contact between the concave meniscus surface and the NIRS-probe during measurements. However, we believe this effect is minor on the study outcomes, as measurements were performed in dark room where it was easy to ascertain if light was escaping between the probe tip and sample surface. A dedicated probe contact study would be necessary in the future, especially regarding measurements to be performed within the joint space during arthroscopy. During arthroscopy it may be more challenging to ensure proper contact due to limited space and visual guidance. This challenge could be potentially solved using convex probe tip that matches the concave curvature of the meniscus surface or by creating an automated algorithm for detecting suboptimal spectral acquisitions resulting from poor probe-sample contact. This is an issue that warrants future study. Another important aspect warranting future study is related to defining the threshold values of the reference parameters, which would classify the current condition of the meniscus. From clinical point of view, we believe that NIRS assessment of meniscal quality could provide important information that is currently unavailable through any other surgical means. This would be useful for helping the clinician to decide whether meniscal repair would benefit a patient, considering the poor success rate of current meniscal repair strategies.^19^,^29^ With the availability of more information on the state of the meniscus during the surgery, the outcome of repair surgery could be greatly improved.

In conclusion, the potential of NIRS for mapping meniscus biochemical composition (water, UA, and HP) using second and third optical window wavelength ranges was investigated. The present results indicate that NIRS could enable rapid mapping of degenerative changes in meniscus biochemical constituents, thus providing means for quantitative arthroscopic assessment of meniscus. However, further studies are required for the method to achieve accuracy required in clinical environment as a supportive tool for decision making.
